# Sushi domain-containing protein 4 binds to epithelial growth factor receptor and initiates autophagy in an EGFR phosphorylation independent manner

**DOI:** 10.1186/s13046-022-02565-1

**Published:** 2022-12-29

**Authors:** Konstantinos S. Papadakos, Alexander Ekström, Piotr Slipek, Eleni Skourti, Steven Reid, Kristian Pietras, Anna M. Blom

**Affiliations:** 1 Division of Medical Protein Chemistry, Department of Translational Medicine, Lund University, Inga Maria Nilsson’s street 53, 214 28 Malmö, Sweden; 2 Division of Translational Cancer Research, Department of Laboratory Medicine, Lund University, Lund, Sweden

**Keywords:** SUSD4, EGFR, AMPKα1, Autophagy

## Abstract

**Background:**

Sushi domain-containing protein 4 (SUSD4) is a recently discovered protein with unknown cellular functions. We previously revealed that SUSD4 can act as complement inhibitor and as a potential tumor suppressor.

**Methods:**

In a syngeneic mouse model of breast cancer, tumors expressing SUSD4 had a smaller volume compared with the corresponding mock control tumors. Additionally, data from three different expression databases and online analysis tools confirm that for breast cancer patients, high mRNA expression of SUSD4 in the tumor tissue correlates with a better prognosis. In vitro experiments utilized triple-negative breast cancer cell lines (BT-20 and MDA-MB-468) stably expressing SUSD4. Moreover, we established a cell line based on BT-20 in which the gene for EGFR was knocked out with the CRISPR-Cas9 method.

**Results:**

We discovered that the Epithelial Growth Factor Receptor (EGFR) interacts with SUSD4. Furthermore, triple-negative breast cancer cell lines stably expressing SUSD4 had higher autophagic flux. The initiation of autophagy required the expression of EGFR but not phosphorylation of the receptor. Expression of SUSD4 in the breast cancer cells led to activation of the tumor suppressor LKB1 and consequently to the activation of AMPKα1. Finally, autophagy was initiated after stimulation of the ULK1, Atg14 and Beclin-1 axis in SUSD4 expressing cells.

**Conclusions:**

In this study we provide novel insight into the molecular mechanism of action whereby SUSD4 acts as an EGFR inhibitor without affecting the phosphorylation of the receptor and may potentially influence the recycling of EGFR to the plasma membrane.

**Supplementary Information:**

The online version contains supplementary material available at 10.1186/s13046-022-02565-1.

## Background

Sushi domain-containing protein 4 (SUSD4) is an unexplored protein described in only a handful of publications. SUSD4 is a transmembrane protein with 4 extracellular “sushi” domains, also known as complement control protein (CCP) domains. Accordingly, we previously showed that SUSD4 can inhibit the classical and alternative complement pathways [1]. Unpredictably, we also found that breast cancer patients with SUSD4 expressing tumors had improved overall survival and less recurrence compared to those with non-expressing tumors [2]. Additionally, SUSD4 expression was corelated with more differentiated tumors and formed fewer lymph node metastases. In vitro, triple negative breast cancer cell lines expressing SUSD4 migrated slower and invaded through Matrigel with lower efficacy [2]. These observations indicate that SUSD4 acts as a tumor suppressor in breast cancer but by an unknown molecular mechanism. Interestingly, SUSD4 was also found to be expressed by tumor infiltrating T cells [2].

Epithelial Growth Factor Receptor (EGFR), on the other hand, is one of the best studied receptor tyrosine kinases and plays a critical role in the homeostasis of the cell, as well as for the progression of most cancer types. Especially, mutations that lead to its constitutive activation are corelated with poorer survival of cancer patients. In lung cancer, EGFR activation is crucial for the progression of the tumor and the response to specific drug treatments [3]. Unstimulated EGFR is autoinhibited in the plasma membrane, remaining in a monomeric state. Upon stimulation with EGF, the EGFR forms homodimers, which is followed by structural rearrangements of the intracellular domain leading to allosteric activation of the EGFR kinase domain. This results in trans-autophosphorylation of the intracellular part and intracellular signaling [4]. Recently, a kinase-independent noncanonical EGFR function was identified [5]. EGFR knockout in mice is lethal [6–8] but kinase-dead EGFR knock-in mice were viable with mild defects [9], suggesting kinase-independent functions of the receptor. Stress conditions (UV radiation, cisplatin, tumor necrosis factor α) trigger endocytosis of the EGFR in a ligand-independent but clathrin and p38-MAPK mediated manner [10]. After internalization, the EGFR is present in a specific population of multivesicular bodies different from those that enclose EGFR stimulated with a ligand and remains trapped there without being degraded in intraluminal vesicles [11]. The process can be reversed when p38-MAPK activity is inhibited [12].

Autophagy is a mechanism of recycling cellular compartments or molecules and allows cells to adapt faster to extracellular changes. In cancer, autophagy is dysregulated and plays a crucial role in chemoresistance. Recent studies identified EGFR as an important regulator of autophagy. Under normal conditions and ligand activation of EGFR, autophagy is repressed directly by inhibiting Beclin-1 [13] in an EGFR-mediated phosphorylation dependent manner. Autophagy is also suppressed indirectly, by activating Akt, which regulates autophagy both by phosphorylating mTOR, an inhibitor of autophagy activation [10], and by inhibiting the activation of AMPKα1, which can regulate the initiation of autophagy directly through phosphorylation of ULK1. Upon starvation, the inactive EGFR is internalized and accumulates in LAPTM4B (lysosomal-associated protein transmembrane 4 beta)- positive vesicles where it binds to Rubicon, an inhibitor of Beclin-1 and dissociates the interaction between the two molecules, resulting in Beclin-1 activation and autophagy initiation [14].

The aim of the study was to investigate the molecular mechanism by which SUSD4 inhibits tumor progression. We first confirmed that SUSD4 acts as tumor suppressor in vivo in a syngeneic mouse model. Onwards, we found that SUSD4 interacts with the EGFR and that both colocalize with endosomal markers. Additionally, we found that SUSD4 promotes initiation of autophagy by a mechanism that requires the expression of EGFR but is independent of its phosphorylation. While the expression of SUSD4 did not affect EGFR downstream signaling or Beclin-1 dissociation from Rubicon, it affected the activation of the tumor suppressor LKB1 and AMPKα1. Finally, SUSD4 expression resulted in an altered phosphorylation status of ULK1, Atg14 and Beclin-1 that favors the initiation of autophagy.

## Methods

### Cell culture and generation of stable expressing cell lines

Breast cancer cell lines BT-20 (ATCC), HS-578 T and MDA-MB-468 (DSMZ) were obtained directly from vendors and maintained in culture for a limited number of passages. The culture media were composed of DMEM high glucose (BT-20 and HS-578 T) or RPMI 1640 (Hyclone), supplemented with 10% fetal bovine serum (Thermo Fisher Scientific), penicillin and streptomycin (Hyclone). Cells were regularly tested for *Mycoplasma* contamination. For protein expression, cells were transfected with eukaryotic expression plasmids containing the appropriate cDNA of the gene (Table S1) using Lipofectamine 3000. After antibiotic (G418, 1 mg/ml) selection, cells were sorted at the flow cytometry core facility of Lund University Diabetes Center, using FACSAria Fusion instrument (BD) and antibody labeling (α-SUSD4 and α-rabbit Alexa Fluor Plus 488).

### Migration and invasion assay

For the migration assay, insert with a pore size of 8.0 μM diameter were used jointly with the accompanying 24-well plates (Corning-Falcon). In the insert, 5 × 10^4^ cells were seeded in a total volume of 250 μl of culture media containing 1% FBS. In the 24-well plate, 750 μl of the medium with 10% FBS was used. After 48 h incubation, remaining non-migrating cells were removed from the upper chamber, migrated cells were fixed with 4% PFA (Merck), stained with 0.5% crystal violet, and images captured with EVOS XL Core digital inverted microscope (Thermo Fisher Scientific). The total number of migrated cells was quantified by the QuPath-software. The same procedure was followed for the invasion assay, except for the use of BioCoat matrigel invasion chambers with 8.0 μm pores (Corning).

### Detection of protein expression, interactions and deglycosylation

Cells were lysed with RIPA buffer (150 mM NaCl, 10 mM Tris-HCl [pH 7.2], 0.1% SDS, 1% Triton X-100, 1% deoxycholate) supplemented with Halt protease and phosphatase inhibitors (Thermo Fisher Scientific). Protein concentration was quantified with BCA protein assay kit (Thermo Fisher Scientific) and equal amounts of total protein was loaded and separated by Mini-PROTEAN or criterion TGX Gels (Bio-Rad). Proteins were transferred to PVDF membranes using Trans-Blot Turbo Transfer System (Bio-Rad) and immunodetected using the appropriate primary and secondary antibodies (Table S3). Signal was detected with Immobilon Western Chemiluminescent HRP Substrate (Merck) and ChemiDoc imaging system (Bio-Rad). For the activation of AMPKα1, AICAR (S1802) and A-769662 (S2697) from Selleckem were used. Proteome profiler antibody arrays (Human Phospho-Kinase Array & Human XL Oncology Array) purchased from R&D systems were performed following the manufacturer’s instructions. For detection of protein interactions using ELISA, cells were lysed with NP40 buffer (150 mM NaCl, 50 mM Tris-HCl [pH 7.5], 1% NP40) supplemented with Halt protease and phosphatase inhibitors (Thermo Fisher Scientific). The appropriate capturing antibody (total amount EGFR: 200 ng, and PDGFR: 100 ng) coated overnight in PBS in Nunc MaxiSorp (Thermo Fisher Scientific) plates and equal amounts (total amount EGFR: 600 μg, and PDGFR: 1100 μg) of total cell lysates incubated overnight at 4 °C. The interacting protein was detected with anti-SUSD4 (total amount: 130 ng) and the suitable secondary anti-rabbit antibody (Table S2). Signal was detected by developing with TMB substrate (Kem-En-Tec Nordic) and measuring with Cytation 5 (Biotek). Deglycosylation of SUSD4 was achieved by using either the Protein Deglycosylation Mix II (NEB) or PNGase F (NEB) according to the manufacturers’ instructions. For immunoprecipitation 500 μg of total protein NP-40 lysates, were incubated overnight with 5 μg anti-SUSD4 antibody. The protein complexes were precipitated with 50 μl of protein G Dynabeads (Thermo Fisher Scientific). Finally, the beads were washed three times with NP40 buffer, and the proteins were eluted with 40 μl loading buffer.

### EGFR assays

Isolation of different fractions of cell compartments was performed with Subcellular Protein Fractionation Kit for Cultured Cells (Thermo Fisher Scientific) according to the manufacturer’s instructions. For EGFR degradation assay, 7 × 10^5^ cells were seeded in 6-well plates. After 24 h the medium was removed and replaced with serum-free medium. Following 6 h incubation, the medium was replaced again with new serum-free medium containing 50 μg/ml Cycloheximide (Sigma). Thereafter, the cells were stimulated with 25 ng/ml of EGF (Sigma) for 2 h and total cell lysates were collected at specific time points with RIPA buffer.

### Syngeneic mouse model

The 4 T1-Luc2 mouse breast cancer cells (ATCC) were harvested with Versene (Thermo Fisher Scientific), counted, washed two times in PBS, and resuspended in PBS. The 4 T1-Luc2 cells that recombinantly express mSUSD4 tagged on the N-terminus with FLAG peptide, or corresponding mock control cells (50 μl) were transplanted into the fourth inguinal mammary fat pad of 8-weeks-old female BALB/cJRj mice (10 mice/group; JanvierLabs). The tumors were measured once weekly using a caliper and tumor volume was estimated using the equation: (D x d^2^ x π), where D and d are the major and minor diameters. The experiment was terminated when at least one mouse reached the ethical limit for tumor volume. The experiments were approved by the local Ethical Committees for Animal Experimentation in Lund (4349/2020).

### Confocal microscopy and PLA assay

Breast cancer cells were seeded in 12-well slides with removable chambers (Ibidi). Cells were fixed with 4% paraformaldehyde, permeabilized (PBS, 0.1% Triton X-100), non-specific binding blocked (PBS, 0.1% Triton X-100, 3% BSA) and incubated with primary antibodies for 1 h. The interactions between proteins were then detected using Duolink kit (Sigma) or NaveniFlex (Navinci), following the manufacturers’ instructions. For LC3B puncta visualization, 1 × 10^3^ cells were seeded in 8-well slides with removable chambers (Ibidi). The next day, cells were transfected with 100 ng of pSELECT-GFP-LC3 (Invivogen) using Lipofectamine 3000 following manufacturer’s instructions. After 24 h, the culture medium was replaced and the cells were left to recover for 24 h before treating them with 50 μM Chloroquine (Sigma) for 3 h. The Premo Autophagy Tandem Sensor RFP-GFP-LC3B Kit (Thermo Fisher Scientific) was used according to the manufacturer’s instructions. Detection of vesicles was performed by immunofluorescence staining in three steps to avoid non-specific signal. In the first step, primary antibodies targeting vesicle markers and EGFR were used. Subsequently, we used secondary antibodies targeting the primary antibodies. In the last step, the cells were incubated with an antibody targeting SUSD4 directly labeled with AlexaFluor 488. Images were captured by ZEISS LSM 800 with Airyscan and analyzed with ImageJ software.

### CRISPR-Cas9 knockout cell lines

Targets for sgRNA were detected with CHOPCHOP online tool (Table S1). Oligonucleotides were produced by Eurofins genomics and cloned into pSpCas9(BB)-2A-Puro (PX459) V2.0 which was a gift from Feng Zhang (Addgene plasmid # 62988; http://n2t.net/addgene:62988; RRID: Addgene 62,988), following published method [15]. Genome editing was detected by PCR reaction and confirmed by western blot analysis. Two EGFR knockout and two wild type clones were transfected with pCDNA3 containing cDNA for SUSD4 and at least three clones were kept per EGFR knockout or wild type clone.

### Mutagenesis and transient transfection

Point mutations in the genes encoding SUSD4 and EGFR were introduced with QuikChange II site-directed mutagenesis kit (Agilent) according to manufacturer’s instruction. Deletions of CCP regions in the extracellular domain of SUSD4 were constructed using the same kit but with a modified protocol [16]. Primers used in the mutagenesis reactions are described in Table S2. Transient transfection was performed with Lipofectamine 3000 (Thermo Fisher Scientific) according to manufacturer’s instruction in 6-well plates containing 6 × 10^5^ cells per well. After 48 h, the cells were lysed with RIPA buffer and total cell protein lysates were collected. The EGFR-GFP was a gift from Alexander Sorkin (Addgene plasmid # 32751; http://n2t.net/addgene:32751; RRID:Addgene_32,751) [17]. The pcDNA6A-EGFR ECD (1–644) was a gift from Mien-Chie Hung (Addgene plasmid # 42666; http://n2t.net/addgene:42666; RRID: Addgene_42,666) and the pcDNA6A-EGFR ICD (645–1186) was a gift from Mien-Chie Hung (Addgene plasmid # 42667; http://n2t.net/addgene:42667; RRID: Addgene_42,667) [18].

### Statistical analyses

Graphs and statistics were prepared and calculated with Prism 8 statistical software. When two groups were compared, Student’s t-test was applied for calculating the *p* value. When three or more groups were compared for one variable, one-way analysis of variance (ANOVA) was used while two-way analysis of variance (ANOVA) was used when comparing two or more variables. Normal distribution was assumed for all statistical tests.

## Results

### SUSD4 expression inhibits tumor growth in a syngeneic mouse model

**Fig. 1:**
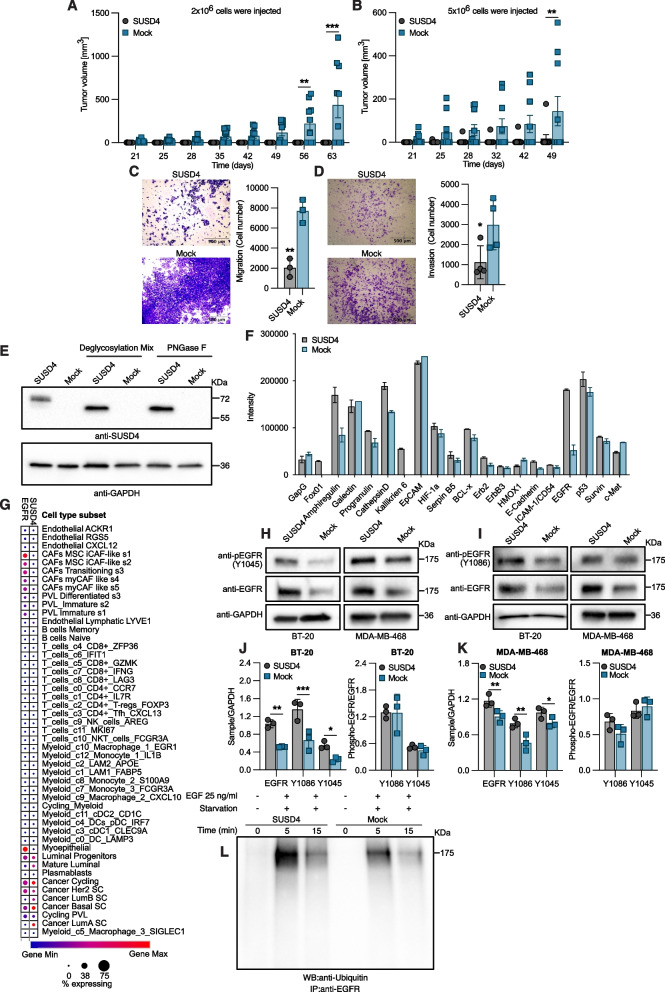
SUSD4 expression suppresses tumor growth and affect EGFR dynamics. Tumor growth monitored in a syngeneic mouse model. 4 T1-Luc2 cells stably expressing mouse SUSD4, or mock control cells were injected into the mammary fat pad of BALB/c mice (*n* = 10 mice per group). In first experiment (**A**) 2 × 10^6^ cells were injected per mouse and in the second (**B**) 5 × 10^6^ cells were injected per mouse (Error bars represents standard error of the mean). (**C**) Migration and invasion assay (**D**) of 5 × 10^4^ 4 T1-Luc2 cells incubated for 48 h under serum. **(E)** Immunodetection of SUSD4 from lysates of BT-20 cells stably expressing human SUSD4 or mock control cells treated with deglycosylation enzymes to assess SUSD4 N-glycosylation status. **(F)** Protein profile comparison between SUSD4-expressing BT-20 cells and mock control cells using the Human XL Oncology Array (*n* = 2 technical repeats). **(G)** Expression of SUSD4 and EGFR, independent of each other, in individual cell types present in breast cancer tumors. Representative immunoblots depicting total EGFR levels and phospho-EGFR at Tyr1045 **(H)** or Tyr1086 **(I)** in SUSD4-expressing BT-20 or MDA-MB-468 cells and corresponding mock control cells. Densitometric analysis of total EGFR and phospho-EGFR levels together with the ratio of phosphorylated EGFR to total EGFR in BT-20 cells **(J)** and MDA-MB-468 cells **(K). (L)** EGFR ubiquitination assessed by immunoprecipitation of EGFR in serum-starved EGF-treated BT-20 cell expressing SUSD4 or mock control cells followed by immunoblot detection of ubiquitin. All results were repeated in at least three independent biological experiments unless otherwise specified. The *p*-value was estimated with t test when two groups were compared and with Two-way ANOVA test when two or more groups were compared for two independent parameters *p ≤ 0.05, **p ≤ 0.01, ***p ≤ 0.001 and ****p ≤ 0.0001

We previously demonstrated that the expression of SUSD4 by tumor cells was correlated with a better prognosis for breast cancer patients [2]. To further evidence a tumor suppressive effect of SUSD4 in vivo, the murine breast cancer cell line 4 T1-Luc2 stably expressing mouse SUSD4-FLAG or mock control cells were transplanted into BALB/c mice. The previously identified complement inhibitory function of SUSD4 could theoretically be beneficial for the tumor [1]. As such, the rationale behind the choice of a syngeneic model was to study the effect of SUSD4 expression in model with non-aberrant immune system. Two doses of cells (2 × 10^6^ or 5 × 10^6^) were injected in the mammary fat pad and tumor growth was then monitored. While no tumors could be detected in the group transplanted with 2 × 10^6^ SUSD4-expressing cells, seven out of 10 mice transplanted with mock control cells developed tumors and a significant difference in tumor volume between the two groups was observed 56 days post transplantation (Fig. 1A). In contrast to the four out of 10 mice transplanted with 5 × 10^6^ mock control cells that developed tumors, only one mouse out of 10 transplanted with 5 × 10^6^ SUSD4-expressing 4 T1-Luc2 cells developed a tumor and a significant difference in tumor volume between the two groups could be observed after 7 weeks (Fig. 1B). The syngeneic model thus provides further support for a tumor suppressive effect of SUSD4. Moreover, upon in vitro characterization, 4 T1-Luc2 cells stably expressing mouse SUSD4-FLAG showed a lower migratory capacity (Fig. 1C) and invasiveness through Matrigel (Fig. 1D) compared to control mock cells. These results are in accordance with previous observations in human breast cancer cell lines [2].

### Expression of SUSD4 affects cellular EGFR levels

The triple negative breast cancer cell line BT-20 was stably transfected with a vector encoding human SUSD4 or a mock control vector. Based on the amino acid sequence, the estimated molecular weight of SUSD4 is 54 kDa, yet consistent with previous results [2], SUSD4 could be detected by western blot at approximately 70 kDa. SUSD4 is predicted by the NetNglyc server to have four potential N-glycosylation sites in its extracellular domain. After treatment of protein lysates of SUSD4-expressing BT-20 cells and mock control cells with PNGase F or Protein Deglycosylation Mix II, the former cleaving only N-linked oligosaccharides whereas the latter is also cleaving some O-linked oligosaccharides, the molecular weight of SUSD4 decreased (Fig. 1E), indicating that the protein carries N-linked oligosaccharides.

As SUSD4 is a poorly described protein, a broad screen of proteins differentially expressed in the presence of SUSD4 was employed to identify its underlying tumor-suppressive mechanism in breast cancer. The Human XL Oncology Array enabled for a protein profile comparison between the SUSD4-expressing BT-20 cells and mock control cells. This revealed a marked elevation of FoxO1 (Forkhead Box O1), Amphiregulin, Cathepsin D and Kallikrein 6 (Fig. 1F&S1A) levels in cells expressing SUSD4. Of note, the largest difference between the two groups could be seen in the upregulation of EGFR in SUSD4-expressing cells. Interestingly, Amphiregulin is a ligand for the EGFR [19] while FoxO1 is a downstream inhibitor of EGFR signaling [20]. To gain more insight into the connection between SUSD4 and EGFR we assessed their expression in individual cell types present in breast cancer using data from the Broad Institute Single Cell Portal [21]. Both proteins were highly expressed by breast cancer tumor cells such as luminal progenitor cells, cycling cancer cells, Her2 enriched cancer cells and basal-like breast cancer cells (Fig. 1G). Cancer associated fibroblasts in turn expressed EGFR but not SUSD4.

To further confirm the results obtained from the protein array, the expression of amphiregulin and EGFR in the SUSD4-expressing BT-20 cells and mock control cells was assessed by quantitative real-time PCR (Fig. S1B). No significant differences could be seen, indicating that the observed differences at protein level were not due to alterations in mRNA expression. The difference in EGFR at protein level indicated by the array was thereafter verified by western blot while simultaneously assessing receptor phosphorylation at Tyr1086 and Tyr1045 (Fig. 1H&I). Both BT-20 cells and MDA-MB-468 cells stably expressing human SUSD4 displayed higher levels of EGFR compared to the corresponding mock control cells. Moreover, SUSD4-expressing cells had higher levels of phosphorylated EGFR at both Tyr1086 and Tyr1045, but this was likely a mere consequence of higher EGFR levels rather than increased activation as no difference in the ratio of phosphorylated EGFR to total EGFR could be observed (Fig. 1J&K).

To then assess if the excess EGFR in SUSD4-expressing cells is plasma membrane-associated or present intracellularly, cell surface proteins of MDA-MB-468 cells expressing or lacking SUSD4 were biotinylated and separated from the intracellular protein fraction. The great majority of SUSD4 was revealed to be present intracellularly and, additionally, the SUSD4-expressing MDA-MB-468 cells had higher level of EGFR in both compartments (Fig. S1C). To address a potential effect on EGFR trafficking, receptor ubiquitination was investigated as it is involved in EGFR internalization. EGFR immunoprecipitation and western blot analysis of receptor ubiquitination in serum-starved EGF treated BT-20 cells expressing SUSD4 or mock control cells proved the EGFR to be ubiquitinated faster and to a greater extent in SUSD4-expressing cells (Fig. 1L). Next, a possible effect of SUSD4 expression on EGFR degradation was investigated by treating serum-starved cells with EGF prior to western blot analysis of EGFR levels. However, the BT-20 SUSD4 and mock control cells exhibited a similar decrease in EGFR levels, thus indicating that the expression of SUSD4 does not affect EGFR degradation (Fig. S1D&E).

### SUSD4 expression promotes autophagy

**Fig. 2:**
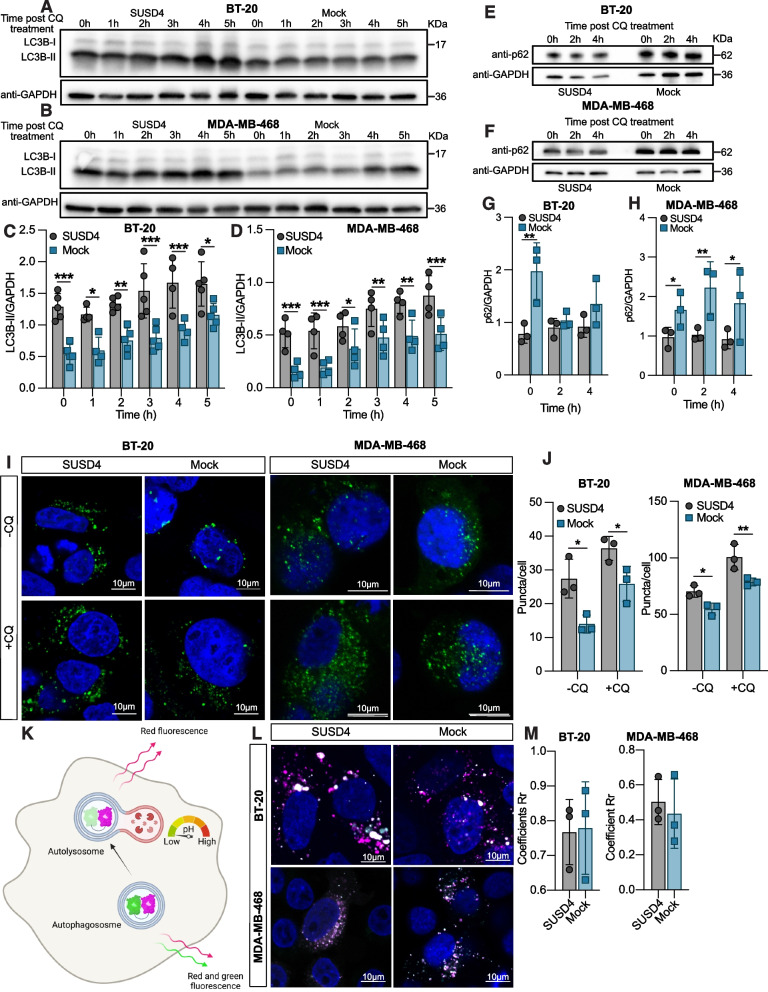
SUSD4 expression leads to increased autophagic flux. Autophagy levels SUSD4-expressing BT-20 cells **(A **and** C)** or MDA-MB-468 cells **(B **and **D)** and corresponding mock control cells treated with chloroquine for up to 5 hours. Representative immunoblots **(A **and** B)** and densitometric analysis **(C **and** D)** of LC3B-II levels in SUSD4-expressing cells and mock control cells for each cell line and chloroquine treatment time. Autophagy levels depicted by p62 levels in SUSD4-expressing BT-20 cells **(E **and** G)** or MDA-MB-468 cells **(F **and** H)** and corresponding mock control cells treated with chloroquine for 2- or 4 hours. Representative immunoblots and densitometric analysis of p62 levels in BT-20 cells **(E **and** G)** and MDA-MB-468 cells **(F **and** H)**. LC3B puncta in SUSD4-expressing BT-20 cells or MDA-MB-468 cells and corresponding mock control cells expressing GPF-tagged LC3B visualized by confocal microscopy. Representative images showing LC3B puncta (green) and DAPI (blue) **(I)** as well as quantified number of puncta per cell **(J)** in untreated cells and cells treated with chloroquine for 3 hours (Every data point represents the average of puncta per cell from at least 10 cells per sample). Assessment of autophagosome-lysosome fusion in BT-20 cells or MDA-MB-468 cell expressing SUSD4 and corresponding mock control cells using the Premo Autophagy Tandem Sensor RFP-GFP-LC3B **(K),** created with BioRender.com. Representative images **(L)** and quantified colocalization coefficient **(M)** of magenta- and green-stained vesicles. (Every data point represents the average of at least 10 cells per sample). All results were repeated in at least three independent biological experiments. The p-value was estimated with t test when two groups were compared and with Two-way ANOVA test when two or more groups were compared for two independent parameters *p ≤ 0.05, **p ≤ 0.01, ***p ≤ 0.001 and ****p ≤ 0.0001

The EGFR plays a well-characterized role in the regulation of autophagy [10, 14] and therefore we hypothesized that SUSD4 may also influence this degradative pathway. To study the effect of SUSD4 expression on autophagy we treated cells with the lysosomotropic agent chloroquine, which inhibits autophagosome fusion with lysosomes by raising the lysosomal pH. Consequently, this leads to an accumulation of the well-established marker for autophagy LC3B-II. Following chloroquine treatment for up to 5 hours, LC3B-II levels assessed by western blot were compared between SUSD4-expressing BT-20 (Fig. 2A&C) or MDA-MB-468 cells (Fig. 2B&D) and corresponding mock control cells. Strikingly, both BT-20 cells and MDA-MB-468 cells expressing SUSD4 had significantly higher LC3B-II levels than the mock control cells even absent chloroquine exposure. Onwards, the LC3B-II levels increased with the chloroquine treatment time but remained significantly higher in the SUSD4-expressing cells, indicating a clear effect of SUSD4 on autophagy. The same phenomenon was also observed for the triple negative mouse breast cancer cell line 4 T1-Luc2 stably expressing mouse SUSD4-FLAG, which had been treated with chloroquine for 3 hours (Fig. S1F). Furthermore, LC3B-II levels were also evaluated in the luminal A cell lines T47D and MCF-7 stably expressing SUSD4. The T47D cells expressing SUSD4 had significantly higher LC3B-II levels after 2 hours of chloroquine treatment compared to the mock control cells (Fig. S1G). Expression of SUSD4 in MCF-7 did not seem to have effect on LC3B-II levels (Fig. S1H). Notably, there are large differences in EGFR expression levels between the used triple-negative and luminal A cell lines according to GOBO database [22].

This prompted subsequent assessment of SUSD4’s effect on autophagy using p62 as a marker. As a substrate for autophagy, p62 levels decline in the cell upon induction of autophagy while accumulating when the autophagic flux is inhibited. SUSD4-expressing BT-20 or MDA-MB-468 cells and mock control cells were treated with chloroquine prior to western blot-analysis of cellular p62 levels (Fig. 2E-H). In concurrence with the observations made for LC3B-II, a significant difference indicating higher levels of autophagy in SUSD4-expressing cells was seen. The mock control cells exhibited significantly higher p62 levels initially in both cell lines, a difference that persisted over the course of the chloroquine treatment for the MDA-MB-468 cells.

To gain further confirmation, SUSD4-expressing BT-20 and MDA-MB-468 cells together with the corresponding mock control cells were transfected with a vector encoding GFP-tagged LC3B, allowing for the visualization of LC3B coated vesicles, i.e. autophagosomes, as puncta. Compared to the mock control cells, a significantly higher number of LC3B puncta per cell was observed for the SUSD4-expressing cells with or without a three-hour chloroquine treatment preceding the fluorescent imaging of the cells (Fig. 2I&J).

The significant differences in both the number of LC3B puncta and LC3B-II levels seen between the SUSD4-expressing cells and the mock control cells in the absence of chloroquine, indicate that SUSD4 may play a role in initiating autophagy, but could also be explained by aberrations in the autophagic flux. To address this, the Premo Autophagy Tandem Sensor RFP-GFP-LC3B was used to evaluate the effect of SUSD4 expression on the fusion between autophagosomes and lysosomes. The dual fluorescent labelling of LC3B with the acid-sensitive GFP and acid-insensitive RFP allowed for the membranous compartment where LC3B is localized to be identified. The presence of both GFP and RFP causes autophagosomes to stain yellow while the degradation of the acid-sensitive GFP causes autophagolysosomes to stain red (Fig. 2K). No difference in the colocalization of red- and green-stained vesicles could be seen between the SUSD4-expressing cells and mock control cells (Fig. 2L&M), indicating that the fusion process between autophagosomes and lysosomes is not affected by SUSD4 expression and that SUSD4 likely plays a role in initiating autophagy.

### SUSD4 interacts with growth factor receptors

**Fig. 3:**
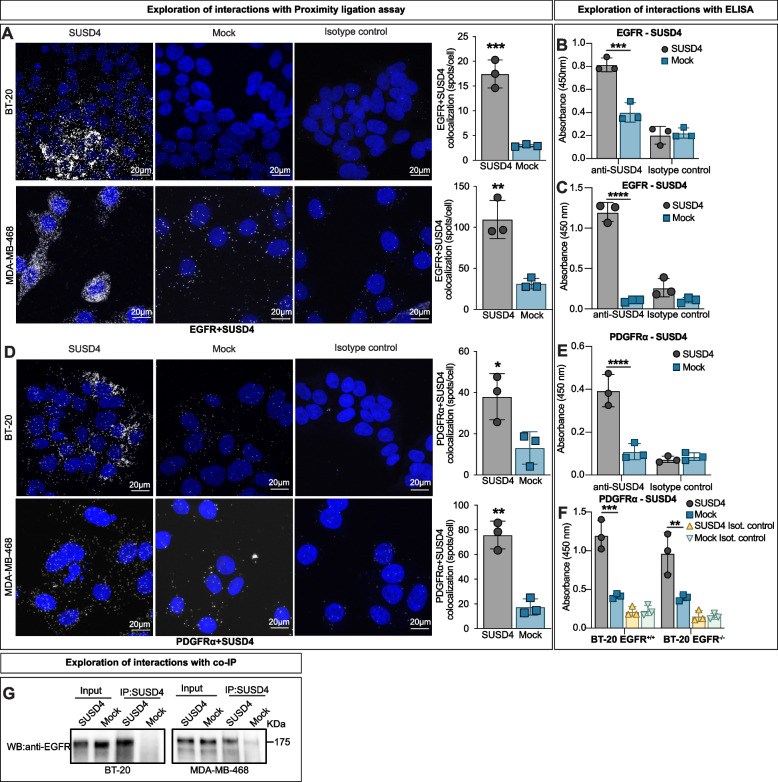
SUSD4 interacts with growth factor receptors.** A** Proximity ligation assay indicating interaction (white spots in maximum intensity projection of z-stack image) between SUSD4 and EGFR in both BT-20 and MDA-MB-468 cells stably expressing SUSD4 (every data point represents the average of at least 10 cells per sample). Sandwich ELISA using lysates of SUSD4-expressing BT-20 **(B)** or MDA-MB-468 cells **(C)** and corresponding mock control cells showing an interaction between SUSD4 and EGFR in both cell lines (*n* = 3 biological repeats in each). **D** Proximity ligation assay indicative of an interaction (white spots in maximum intensity projection of z-stack image) between SUSD4 and PDGFRα in BT-20 cells and MDA-MB-468 cells stably expressing SUSD4 (Every data point represents the average of at least 10 cells per sample). **E** Sandwich ELISA showing an interaction between SUSD4 and PDGFRα in BT-20 cells expressing SUSD4. **F** Same ELISA as in **(E)** but including lysates of CRISPR/Cas9-mediated EGFR knockout cells stably expressing human SUSD4 and corresponding mock control cells. **G** Co-immunoprecipitation of the protein complexes between EGFR and SUSD4 using lysates of the BT-20 and MDA-MB-468 cells stably expressing SUSD4. All results were repeated in at least three independent biological experiments. The *p*-value was estimated with t test when two groups were compared and with Two-way ANOVA test when two or more groups were compared for two independent parameters *p ≤ 0.05, **p ≤ 0.01, ***p ≤ 0.001 and ****p ≤ 0.0001

We next assessed a possible interaction between SUSD4 and EGFR since both are transmembrane proteins. For this purpose, proximity ligation assay and confocal microscopy were employed and pointed to an interaction between SUSD4 and EGFR in both BT-20 and MDA-MB-468 cells (Fig. 3A). Albeit indicative of an interaction, the proximity ligation assay merely shows that the target proteins are in close vicinity of one another. Sandwich ELISA, whereby an antibody against EGFR was used for capturing and an antibody against SUSD4 was used for detection, was therefore used to further evidence an interaction between SUSD4 and EGFR in both cell lines (Fig. 3B&C). Additionally, SUSD4 was assessed for its ability to interact with another growth factor receptor, PDGFRα. By using the same approach, SUSD4 was found to interact with PDGFRα using both proximity ligation assay (Fig. 3D) and sandwich ELISA (Fig. 3E). We next used the CRISPR/Cas9 system to delete EGFR from BT-20 cells (Fig. S1I). These cells were used to test if SUSD4 interacts independently with PDGFRα or if a heterotrimeric complex with the EGFR is present. Although the presence of a heterotrimeric complex was not disproven, SUSD4 was found to interact directly with PDGFRα in cells lacking EGFR using sandwich ELISA (Fig. 3F). Moreover, the interaction between SUSD4 and EGFR was evident upon co-immunoprecipitation (Fig. 3G).

### Effect of SUSD4 on autophagy depends on its interaction with the EGFR

**Fig. 4:**
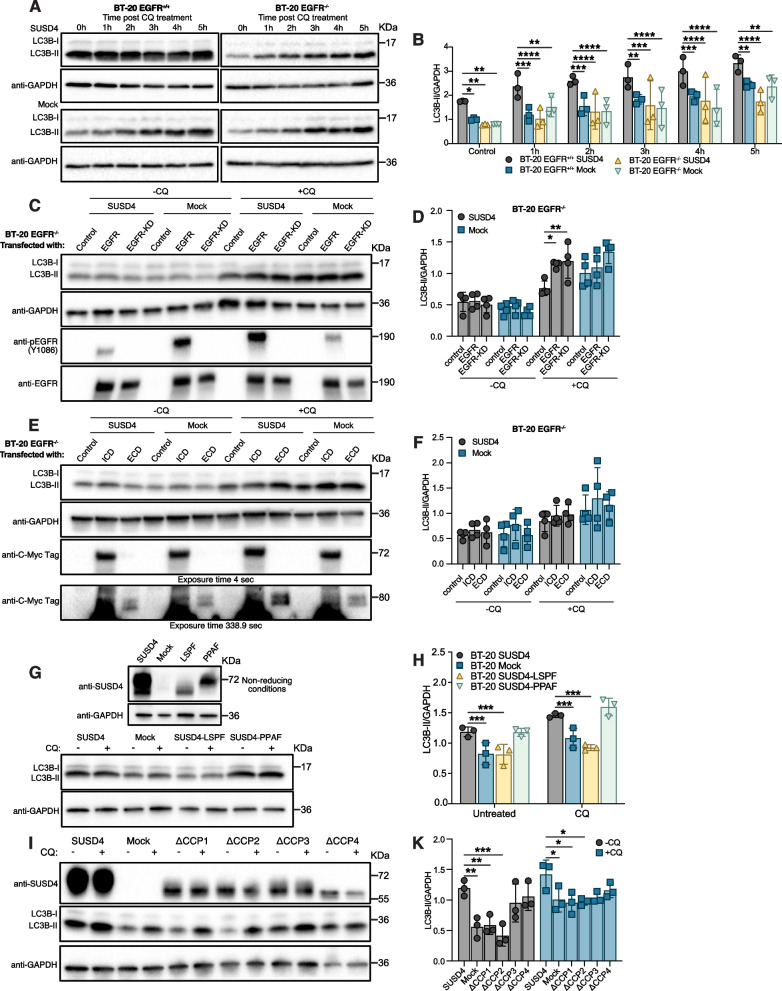
SUSD4’s effect on autophagy depends on its interaction with the EGFR. Autophagy in SUSD4-expressing BT-20 cells and mock control cells in addition to CRISPR/Cas9-mediated EGFR knockout BT-20 cells stably expressing SUSD4 and corresponding EGFR knockout mock control cells. Cells were treated with chloroquine for up to 5 hours and LC3B-II levels were assessed by immunoblot. Representative immunoblots **(A)** and densitometric analysis **(B)** of LC3B-II levels at each time-point. Total EGFR or a kinase-dead mutant EGFR was re-introduced in SUSD4-expressing BT-20 EGFR knockout cells and BT-20 EGFR knockout mock control cells. **C** Representative immunoblot verifying the presence of total-, or kinase-dead EGFR as well as showing LC3B-II levels in both untreated cells and cells treated with chloroquine for 4 hours. **D** Densitometric analysis of LC3B-II levels shown in **(C)** (*n* = 4 biological repeats). Representative immunoblots **(E)** and densitometric analysis **(F)** of LC3B-II levels in untreated and chloroquine treated (4 hours) mock control and SUSD4-expressing BT-20 EGFR knockout cells transiently transfected with constructs allowing for the expression of myc-tagged intra- or extracellular domain of EGFR (*n* = 4 biological repeats). The molecular weight of the EGFR ICD and ECD is 72 kDa and 80 kDa, respectively. **G** Autophagy in BT-20 cells expressing SUSD4 phosphorylation mutants SUSD4-LSPF (Y379F) or SUSD4-PPAF (Y414F). Immunoblot of SUSD4 phosphorylation mutants in addition to a representative immunoblot showing LC3B-II levels in untreated cells and cells treated with chloroquine for 3 hours. **H** Densitometric analysis of LC3B-II levels in BT-20 SUSD4 phosphorylation mutants shown in **(G)**. Deletion mutants of extracellular CCP domains of SUSD4 stably expressed in BT-20 cells. Autophagy was evaluated with LC3B western blots **(I)** in the presence or absence of chloroquine and band density relative to GAPDH was plotted **(K)**. All results were repeated in at least three independent biological experiments. The *p*-value was estimated with t test when two groups were compared and with Two-way ANOVA test when two or more groups were compared for two independent parameters *p ≤ 0.05, **p ≤ 0.01, ***p ≤ 0.001 and ****p ≤ 0.0001

The aforementioned CRISPR/Cas9-mediated EGFR knockout BT-20 cells stably expressing SUSD4 and mock control cells were used to assess whether the effect of SUSD4 on autophagy depends on its interaction with the EGFR. Cells were treated with chloroquine for up to 5 hours prior to assessing LC3B-II levels by western blot. Consistent with the previous results, the cells expressing both SUSD4 and EGFR had significantly higher levels of LC3B-II than the EGFR-expressing mock control cells at all time-points (Fig. 4A&B). This effect was abolished in the EGFR knockout cells, which displayed similar LC3B-II levels as the EGFR-expressing mock control cells, regardless of SUSD4-expression, thus showing that SUSD4’s autophagy-promoting effect is mediated through its interaction with the EGFR.

Whether the effect of this interaction on autophagy depends on the receptor’s kinase activity was thereafter investigated. The EGFR KO cells expressing or lacking SUSD4, were transiently transfected with a vector encoding canonical EGFR, a kinase-dead mutant EGFR, or an empty control vector. Following a subsequent chloroquine treatment, LC3B-II levels were assessed by western blot while simultaneously verifying the absence or presence of EGFR and the kinase-dead mutant EGFR in the cells (Fig. 4C&D). No differences in LC3B-II levels could be seen absent chloroquine exposure and the presence of EGFR or the kinase-dead mutant EGFR did not affect LC3B-II levels in cells lacking SUSD4 expression exposed to chloroquine. In contrast, higher LC3B-II levels following a four-hour chloroquine treatment could be seen in the SUSD4-expressing cells transfected with either the canonical EGFR or the kinase-dead mutant, relative to the control cells. Hence, the effect on autophagy that stems from the interaction between SUSD4 and EGFR does not depend on the receptor’s kinase activity.

To test whether SUSD4 promotes initiation of autophagy by interacting with the intra- or extracellular domain of EGFR, the CRISPR/Cas9 mediated EGFR KO cells expressing or lacking SUSD4 were transiently transfected with constructs enabling the expression of Myc-tagged intra- or extracellular domain of EGFR. The expression of Myc-tagged domains of EGFR was confirmed by western blot and LC3B-II levels with or without a preceding chloroquine treatment was assessed (Fig. 4E&F). Irrespective of the expression of SUSD4, no differences in LC3B-II levels could be observed between cells expressing the intra- or extracellular EGFR domain and cells transfected with an empty vector, both in the presence and absence of chloroquine. Thus, the complete EGFR, rather than solely the intra- or extracellular portion of the receptor, is required for SUSD4-mediated promotion of autophagy. Moreover, two phosphorylation sites in the intracellular domain of SUSD4 have been suggested to be important for protein function [23]. Site-directed mutagenesis was used to address the importance of these phosphorylation sites for SUSD4’s ability to influence autophagy. Untreated and chloroquine-treated BT-20 cells stably expressing SUSD4, SUSD4-LSPF (Y379F) or SUSD4-PPAF (Y414F) were assessed for LC3B-II levels. Western blot analysis revealed a marked decrease in apparent molecular weight for the SUSD4-LSPF mutant while no difference in migration could be seen for the SUSD4-PPAF mutant, relative to the native protein (Fig. 4G). Onwards, the assessment of LC3B-II levels demonstrated a cardinal role for the LSPY phosphorylation site for SUSD4’s ability to promote autophagy (Fig. 4G&H). While no difference in LC3B-II levels, irrespective of chloroquine treatment, could be seen between the SUSD4-expressing cells and the SUSD4-PPAF mutant, significantly lower LC3B-II levels, resembling the mock control cells, were seen for the SUSD4-LSPF mutant, even for the untreated cells. Deletion mutants were constructed to determine, which domains are important for the regulation of autophagic flux. One CCP domain was deleted in each mutated protein, yielding cells expressing SUSD4 ΔCCP1, ΔCCP2, ΔCCP3 or ΔCCP4 (Table S1). Deletion of CCP1 and CCP2 led to impaired induction of autophagy compared to BT-20 wt SUSD4 expressing cells, irrespective of chloroquine treatment (Fig. 4I&K).

### SUSD4 expression leads to activation of signaling complexes promoting autophagy

**Fig. 5:**
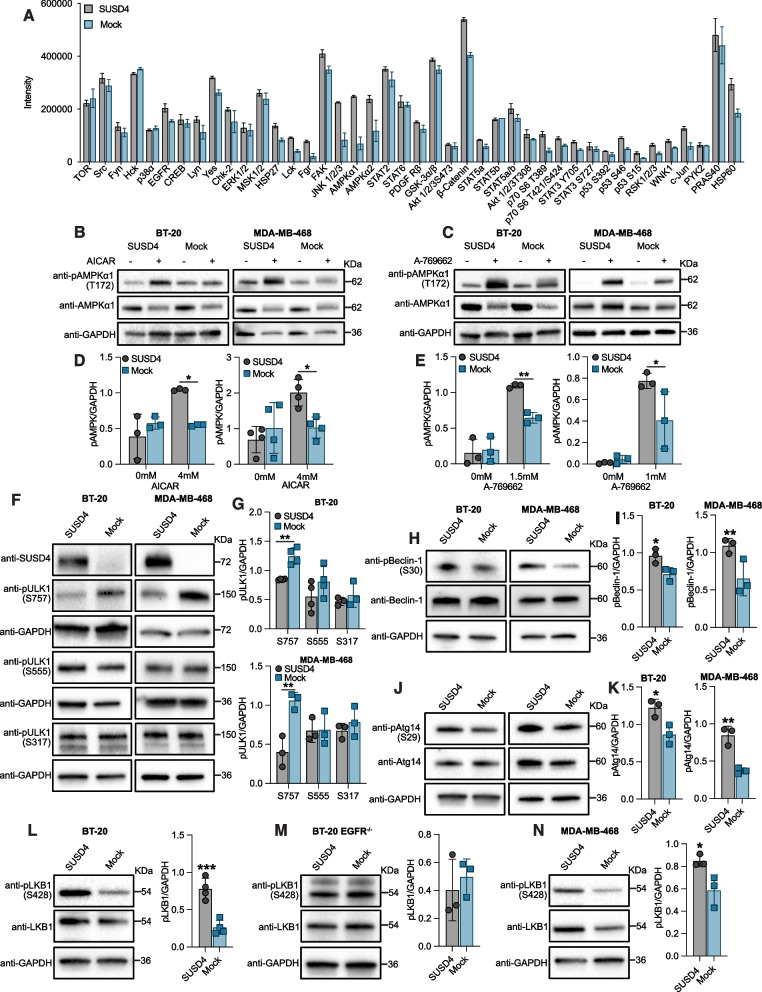
Phosphorylation status of signaling complexes in SUSD4-expressing cells favors autophagy initiation.** A** Comparison in phosphorylation status of a wide range of proteins in SUSD4-expressing BT-20 cells and mock control cells using the Human Phospho-Kinase Antibody Array (*n* = 2 technical repeats). **B-E** Phospho-AMPKα1 levels in SUSD4-expressing BT-20 cells or MDA-MB-468 cells and corresponding mock control cells treated with AICAR **(B **and** D)** or A-769662 **(C **and** E)**. Representative immunoblots **(B **and** C)** and densitometric analysis **(D **and** E)** of phospho-AMPKα1 levels in both treated and untreated cells (*n* = 3 biological repeats for treatment with A-769662 and BT-20 cells treated with AICAR, *n* = 4 biological repeats for MDA-MB-468 cells treated with AICAR). **F and G** Representative immunoblots **(F)** and densitometric analysis **(G)** showing the phosphorylation status of ULK1 at three distinct phosphorylation sites in BT-20 and MDA-MB-468 cells expressing SUSD4 and corresponding mock control cells (*n* = 4 biological repeats for S757 and S555 in BT-20 cells, *n* = 3 biological repeats for S317 in BT-20 cells and all phosphorylation sites in MDA-MB-468 cells). **H **and** I** Representative immunoblot **(H)** and densitometric analysis **(I)** of phospho-activated Beclin-1 in BT-20 cells or MDA-MB-468 cells expressing SUSD4 compared to the respective mock control cells. **J **and** K** Representative immunoblot **(J)** and densitometric analysis **(K)** of phospho-Atg14 levels in BT-20 cells or MDA-MB-468 cells expressing SUSD4 and corresponding mock control cells. **L-N** Representative immunoblots and densitometric analyses showing phospho-LKB1 levels in BT-20 SUSD4 cells and mock control cells **(L)**, BT-20 EGFR knockout cells expressing or lacking SUSD4 **(M)**, and MDA-MB-468 cells expressing or lacking SUSD4. All results were repeated in at least three independent biological experiments. The p-value was estimated with t test when two groups were compared and with Two-way ANOVA test when two or more groups were compared for two independent parameters *p ≤ 0.05, **p ≤ 0.01, ***p ≤ 0.001 and ****p ≤ 0.0001

SUSD4 promotes autophagy through its interaction with the EGFR, but these features are yet to be mechanistically linked. To gain insight into the molecular pathway involved, the Human Phospho-Kinase Antibody Array was utilized as it enabled a comparison in phosphorylation status between the SUSD4-expressing BT-20 cells and mock control cells. The largest differences in phosphorylation status were observed for AMPKα1 (Tyr183), AMPKα2 (Tyr172) as well as JNK1/2/3 (Thr183/Tyr185, Thr221/Tyr223) (Fig. 5A&S1J). Consequently, a role for AMPKα1 was further investigated as it is known to promote autophagy. BT-20 cells and MDA-MB-468 cells stably expressing SUSD4 and corresponding mock control cells were treated with two AMPKα1 activators, AICAR (5-aminoimidazole-4-carboxamide riboside) and A-769662. AMPKα1 activation was subsequently compared by western blot through detection of phospho-AMPKα1 (Tyr172). While no difference in AMPKα1 activation could be seen between the SUSD4-expressing cells and mock control cells for either cell line absent treatment, both AICAR and A-769662 led to significantly higher levels of phospho-activated AMPKα1 in SUSD4-expressing cells compared to mock (Fig. 5B-E).

Upon ligand-induced activation, the EGFR signals through the PI3K-Akt-mTOR axis, leading to Akt-mediated suppression of AMPKα1. As SUSD4-expressing cells demonstrated increased activation of AMPKα1, the modulatory implications of the interaction with SUSD4 on EGFR downstream signaling were evaluated by checking the phosphorylation of Akt at Tyr308 and Ser473, and hence its activation. BT-20 cells expressing SUSD4 exhibited significantly lower levels of phosphorylated Akt at both Tyr308 and Ser473. In contrast, however, no difference in phospho-Akt could be seen between cells expressing SUSD4 and corresponding mock control cells for neither MDA-MB-468 cells nor HS-578 T cells at either phosphorylation site (Fig. S2A). Conversely, MDA-MB-468 cells expressing SUSD4 displayed lower levels of phospho-mTOR (Ser2448) than corresponding mock control cells while no difference in mTOR activation could be seen between BT-20 cells or HS-578 T cells expressing SUSD4 and corresponding mock control cells (Fig. S2B).

While activated AMPKα1 can activate the Unc-51 like kinase-1 (ULK1) through phosphorylation of Ser555 and Ser317 to promote autophagy, mTORC1 can suppress autophagy by phosphorylating ULK1 at Ser757. Comparing the levels of phospho-ULK1 between SUSD4-expressing cells and mock control cells at all three phosphorylation sites revealed significantly lower levels of ULK1 phosphorylated at Ser757 in both BT-20 cells and MDA-MB-468 cells expressing SUSD4, but no difference in ULK1 phosphorylated at Ser555 or Ser317 in either cell line (Fig. 5F&G). Explicitly, SUSD4-expressing cells displayed lower levels of mTORC1-mediated suppression of ULK1 while no difference could be seen in AMPKα1-mediated phospho-activation.

Next, the effect of SUSD4 expression on the activation of complexes involved in autophagosome formation was investigated. Compared to mock control cells, SUSD4-expressing BT-20 and MDA-MB-468 cells exhibited higher levels of activated Beclin-1 as indicated by its phosphorylation at Ser30 (Fig. 5H&I). Similarly, increased phosphorylation and activation of Atg14 could also be observed for BT-20 cells and MDA-MB-468 cells expressing SUSD4 relative to the corresponding mock control cells (Fig. 5J&K). Moreover, LKB1 is an upstream regulator of AMPKα1 that upon activation through phosphorylation at Ser428, can in turn phospho-activate AMPKα1. In BT-20 cells, expression of SUSD4 resulted in increased activation of LKB1 (Fig. 5L), an effect abolished in CRISPR/Cas9-mediated EGFR knockout cells (Fig. 5M). Additionally, increased activation of LKB1 because of SUSD4 expression was further evidenced in MDA-MB-468 cells (Fig. 5N).

### Intracellular localization of SUSD4 and EGFR

**Fig. 6:**
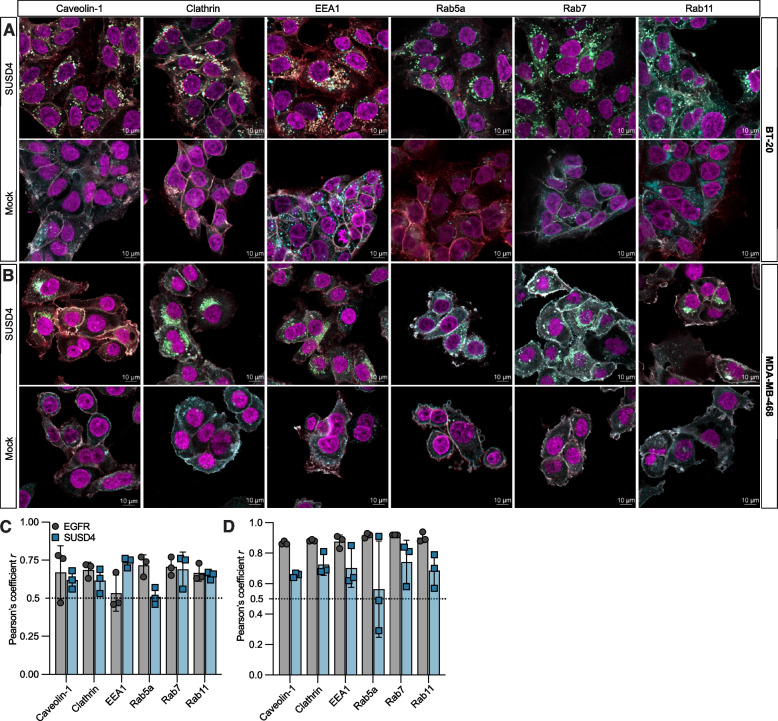
SUSD4 colocalization with endo−/lysosomal markers. Representative merged confocal images showing EGFR (red), SUSD4 (green), endo−/lysosomal markers (blue) and DAPI (magenta) in BT-20 cells **(A)** or MDA-MB-468 cells **(B)** expressing SUSD4 and corresponding mock control cells. Note that the combination of the colors red, green, and blue is presented in white color. Colocalization coefficient for EGFR or SUSD4 with endo−/lysosomal markers quantified in BT-20 cells **(C)** and MDA-MB-468 cells **(D)** (Every data point represents the average of at least 10 cells per sample). All results were repeated in at least three independent biological experiments. Pearson’s correlation coefficient r where a minimal r value of 0.5 (dotted line) indicates significant colocalization

In addition to the plasma membrane, both SUSD4 and EGFR were found intracellularly (Fig. S1C). To further investigate the intracellular compartments harboring SUSD4 and EGFR, their colocalization with various endosomal markers was investigated in BT-20 and MDA-MB-468 cells by confocal microscopy (Fig. 6A, B & S3,4). The localization of both SUSD4 and EGFR positively correlated with the localization of caveolin and clathrin-coated vesicles (Fig. 6C&D). Furthermore, while EGFR colocalized with the early endosomal markers EEA1 and Rab5a in MDA-MB-468 cells, a significant colocalization could only be seen with Rab5a in BT-20 cells. Additionally, SUSD4 was found to colocalize with EEA1 in both BT-20 cells and MDA-MB-468 cell, but not with Rab5a in either cell line. Both EGFR and SUSD4 were found to colocalize with the marker for late endosomes, Rab7 and Rab11, a marker for recycling endosomes. The elevated EGFR levels in SUSD4-expressing cells could not be explained by a difference in mRNA expression (Fig. S1B) or receptor degradation (Fig. S1D&E). Therefore, it is possible that SUSD4 plays a role in the recycling of EGFR to the plasma membrane.

In addition to the suppressive effect on autophagy following ligand-induced activation of EGFR and downstream signaling via the PI3K-Akt-mTOR axis, inactive EGFR has been shown to promote autophagy [14]. This pathway entails internalization of inactive EGFR because of serum-starvation, sequestration in endosomes where it can interact with LAPTM4B, which ultimately leads to the dissociation of Beclin-1 from Rubicon and autophagy initiation. To investigate if SUSD4 plays a role in this pathway, proximity ligation assay was employed to assess the interaction between Beclin-1 and Rubicon in the BT-20 cells and MDA-MB-468 cells expressing SUSD4 as well as the corresponding mock control cells. Under normal conditions, no difference in the interaction between Beclin-1 and Rubicon could be observed between the SUSD4-expressing cells and mock control cells in either cell line (Fig. S5A). However, upon serum-deprivation, a decreased level of interaction between Beclin-1 and Rubicon could be seen in SUSD4-expressing BT-20 cells compared to the mock control cells (Fig. S5B). In contrast, no difference could be seen between the MDA-MB-468 cells expressing SUSD4 and the mock control cells (Fig. S5B).

### SUSD4 expression in breast cancer cells correlates with improved patient prognosis

**Fig. 7:**
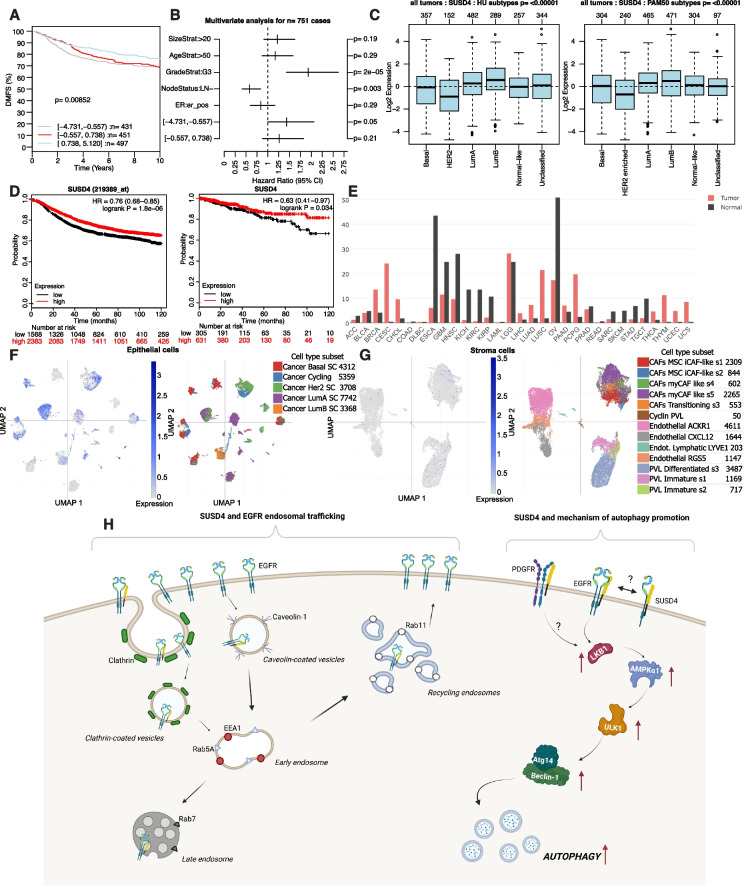
SUSD4 expression data in breast cancer. Expression data for SUSD4 in breast cancer patients obtained from the GOBO database. **A** Kaplan-Meier survival analysis displaying distant metastasis free survival of patients with high expression of SUSD4 (log2 expression 0.738, 5.120), intermediate expression (log2 expression − 0.557, 0.738) or low expression (log2 expression − 4.731, − 0.557). **B** Multivariate analysis indicating low SUSD4-expression as an independent prognostic factor for survival. **C** Expression of SUSD4 in breast cancer subsets. **D** Kaplan Meier survival analysis showing relapse free survival for breast cancer patients with high or low expression of SUSD4 assessed by microarray analysis (left, *n* = 3951) or mRNA sequencing (right, *n* = 936). **E** Data obtained from the Gene Expression Profiling Interactive Analysis database showing the expression of SUSD4 in tumorous tissues compared to paired healthy tissues. Data obtained from the Broad Institute Single Cell Portal showing the expression of SUSD4 in epithelial cells of different breast cancer subtypes **(F)** and in different stromal cells **(G)**. Figure showing a proposed effect of SUSD4 expression in breast cancer cells and its relationship to EGFR and autophagy. In brief, SUSD4 binds to EGFR and initiates autophagy in an EGFR phosphorylation-independent manner. Autophagy initiation is affected by the phosphorylation of SUSD4 at the LSPY site. In addition, CCP1 and 2 in the extracellular part of SUSD4 play a role in autophagy initiation. Onwards, the downstream pathway of LKB1, AMPKa1, ULK1, Atg14 and Beclin-1 is activated, leading to increased autophagic flux. Finally, SUSD4 colocalizes with different endosomal vesicles. SUSD4 and EGFR are highly colocalized with Rab11-positive recycling endosomes indicating a potential effect of SUSD4 on EGFR recycling to the plasma membrane, **(H)** created with BioRender.com

The expression of SUSD4 was previously correlated with a better prognosis for breast cancer patients [2]. Data now obtained from the Gene expression-based Outcome for Breast cancer Online (GOBO) database [22] and assessed by Kaplan-Meier survival analysis further corroborated a positive outcome related to SUSD4 expression (Fig. 7A). Following the division of patients into three groups based on their expression of SUSD4: high expression (log2 expression 0.738, 5.120), intermediate expression (log2 expression − 0.557, 0.738) and low expression (log2 expression − 4.731, − 0.557), a high expression of SUSD4 could be correlated with improved distant metastasis free survival (Log-rank *p* = 0.00852). Furthermore, a low expression of SUSD4 was identified as an independent prognostic factor (*p* = 0.038) using multivariant analysis (Fig. 7B). Looking at the expression of SUSD4 in various subtypes of breast cancer revealed an upregulation in the subtypes luminal A and luminal B, which have a better prognosis (Fig. 7C). Additionally, SUSD4 was found to be downregulated in HER2 enriched breast cancer and unchanged in Normal-like breast cancer.

According to the Kaplan Meier plotter online survival analysis tool [24] for breast cancer patients, a microarray analysis of mRNA expression in patients (*n* = 3951) examined with Kaplan-Meier survival analysis revealed a longer relapse free survival for patients with high expression of SUSD4 (Log-rank p = < 0.0001) (Fig. 7D). The same online tool used for a Kaplan-Meier survival analysis of mRNA sequencing data of breast cancer patients in the pan-cancer project (*n* = 936), further corroborated a longer relapse free survival correlated with high expression of SUSD4 (Log-rank *p* = 0.034) (Fig. 7D). In accordance, patients with a high expression of SUSD4 and upregulated expression of autophagy markers (LC3B or p62) had longer relapse free survival compared with those with low expression of SUSD4 and upregulated expression of autophagy markers (Fig. S5C&D).

Moreover, comparing the expression of SUSD4 in tumorous tissues and matched normal tissues using the Gene Expression Profiling Interactive Analysis (GEPIA) database [25], showed an upregulation of SUSD4 in various cancer types, including breast cancer, while it was downregulated in others, for example ovarian cancer (Fig. 7E). Onwards, using single cell RNA sequencing data obtained from the Broad Institute Single Cell Portal [21], enabled for the expression of SUSD4 in both epithelial cells of various breast cancer subtypes (Fig. 7F) and in different stromal cells (Fig. 7G) to be evaluated. Correlating the level of SUSD4 expression seen in blue to the left in each panel, with the color-annotated breast cancer subsets (Fig. 7F) or color-annotated stromal cell subsets (Fig. 7G), shows that SUSD4 is expressed by breast cancer epithelial cells of the various subtypes of breast cancer, but not by the stromal cells. Hence indicating that SUSD4 is expressed by the cancerous cells rather than the tumor-associated stromal cells.

## Discussion

Many proteins remain unexplored with the vast majority of the publications focused on approximately 2000 of the 19,000 [26] gene products of the human genome. SUSD4 was first identified following genome-wide sequencing studies but has since remained a meagerly researched protein with largely uncharacterized functions. Microdeletions of the chromosomal region harboring the gene has previously been linked to Fryns syndrome [27, 28]. Additionally, SUSD4 is highly expressed in the CNS [1] and prior studies using *SUSD4* knockout mice revealed an abnormal morphology of neuronal cells as well as abnormalities in learning, motor function and behavior in the knockout mice [23]. Furthermore, in addition to its complement-regulatory properties [1], we have previously proposed a tumor suppressive effect of SUSD4 [2], yet the underlying molecular mechanism has not previously been addressed. Here we provide further evidence corroborating a tumor suppressive role of SUSD4 as well as novel mechanistic insight into the role of SUSD4 in breast cancer cells, revealing both interaction partners and an autophagy-promoting effect.

In this study, SUSD4 was found to interact with the tyrosine-kinase receptors EGFR and PDGFRα in triple negative breast cancer cell lines. The two receptors have been found to engage in heterodimers [29, 30] and it could be that a heterotrimeric complex is formed together with SUSD4. Although the interaction between SUSD4 and PDGFRα persisted in CRISPR/Cas9-mediated EGFR knockout cells, thus showing that the interaction between SUSD4 and PDGFRα occurs independent of the EGFR, it does not negate the existence of a plausible heterotrimeric complex. Further investigation is required to determine if SUSD4 interacts with additional tyrosine kinase receptors. In general, breast cancer cell lines were very hard to transfect in a stable manner with SUSD4 and some rapidly lost their expression in culture. Finally, the main cell lines used in this study were those that maintained SUSD4 expression, which may be related to the fact that they also have high expression of EGFR as based on assessment using the GOBO database [22].

Relative to mock control cells, SUSD4-expressing cells had higher protein levels of EGFR despite the lack of alterations in mRNA expression levels or receptor degradation, which together suggests a role for SUSD4 in EGFR recycling. Furthermore, both SUSD4 and EGFR were found to colocalize with various endosomal markers, including Rab11 which is a marker for recycling endosomes. This further points to a role of SUSD4 in EGFR trafficking and recycling. Moreover, no consensus was reached regarding a plausible effect of the interaction with SUSD4 on EGFR downstream signaling. The three breast cancer cell lines analyzed gave inconsistent results in terms of phospho-activated Akt and mTOR levels. This could potentially be explained by yet unidentified cellular mechanisms of SUSD4 and/or inherent differences between the cell lines. The BT-20 cell line reportedly carries a mutation in the *PIK3CA* gene while the MDA-MB-468 cell line carries a mutation in *PTEN* and the HS-578 T cell line has a mutated HRAS [31]. The *PIK3CA* gene encodes a subunit of PI3K, PTEN is a negative regulator of PI3K-Akt signaling [32] and HRAS is a GTPase of the RAS family, which are known to affect signaling through PI3K and Akt [33]. These mutations may cause intrinsic aberrations in signaling along the PI3K-Akt-mTOR axis that explain the inconclusive results pertaining to SUSD4’s effect on EGFR downstream signaling.

We found that SUSD4 promotes autophagy in a strictly EGFR-dependent manner as the effect was abolished in CRISPR/Cas9-mediated EGFR knockout cells. Since the fusion process between autophagosomes and lysosomes was unaffected, SUSD4 presumably plays a role in initiating autophagy. Considering the interaction between SUSD4 and EGFR, the dependence on EGFR for increased autophagic flux and colocalization of both EGFR and SUSD4 with endosomal markers, a hypothesis suggesting that SUSD4 promotes autophagy in a manner resembling that seen upon serum starvation grew forth. Upon serum starvation, inactive EGFR is internalized and accumulates in endosomal compartments where it through its association with LAPTM4B and the Sec5 exocyst subcomplex promotes Beclin-1 dissociation from Rubicon and initiation of autophagy [14]. Additionally, tyrosine kinase inhibitors targeting EGFR, such as Erlotinib and Gefitinib, induce autophagy in an EGFR-dependent manner similar to serum starvation and inhibit receptor phosphorylation [14]. Contrastingly yet similarly, SUSD4 promotes autophagy only in the presence of the EGFR, but without any effect on EGFR phosphorylation status. Despite the plausibility that SUSD4 induces autophagy through the abovementioned pathway, the proximity ligation assay between Beclin-1 and Rubicon did not support this hypothesis, thus suggesting that autophagy is promoted via a different pathway.

Onwards, we found that signaling complexes renowned for their ability to promote autophagy exhibited an altered phosphorylation status favoring initiation of autophagy in SUSD4-expressing cells. This includes the tumor suppressor LKB1, AMPKα1, ULK1, Atg14 and Beclin-1. These complexes exert their roles sequentially to promote autophagy and is subject to extensive crosstalk with the PI3K-Akt-mTOR signaling pathway downstream of the EGFR. However, in the cell lines tested, SUSD4 affect EGFR downstream signaling in a phosphorylation independent manner, suggesting that the autophagy-promoting signaling complexes may be subject to other input. Nevertheless, SUSD4-expressing cells displayed reduced suppression of ULK1 while no difference in phospho-activation of ULK1 could be detected. This, together with the fact that the elevated levels of phospho-activated LKB1 in SUSD4-expressing cells were abolished in EGFR knockout cells, suggests that SUSD4’s effect on autophagy stems from an effect on EGFR downstream signaling in a novel unexplored manner. We revealed a novel molecular mechanism wherein SUSD4 induces the initiation of autophagy through its interaction with EGFR and consequently activation of LKB1, AMPKα1, ULK1, Atg14 and Beclin-1 (Fig. 7H). The initiation of autophagy was independent from EGFR phosphorylation, but it was affected by the phosphorylation of SUSD4 at the LSPY site. Moreover, the first two CCP domains of SUSD4 were important for autophagy initiation. The novel way by which the interaction between SUSD4 and EGFR leads to activation of LKB1 remains to be fully understood.

## Conclusions

In conclusion, we provide further evidence for a tumor suppressive role of SUSD4 and we deciphered the underlying mechanism. Interactions of SUSD4 with growth factor receptors that play well-characterized roles in tumor progression and development were identified in addition to an autophagy-promoting effect. We also propose a potential role for SUSD4 in EGFR trafficking and recycling in breast cancer cells. Further studies are needed to fully elucidate the roles of the novel breast cancer suppressor SUSD4 in both cancerous cells as well as in the brain, where it is highly expressed.

## Supplementary Information

**Additional file 1.**

## Data Availability

All data are available in the main text or the supplementary materials.

## Abbreviations

SUSD4: Sushi domain-containing protein 4
CCP: Complement control protein
EGFR: Epithelial Growth Factor Receptor
PDGFRα: Platelet-derived growth factor receptor alpha
LAPTM4B: lysosomal-associated protein transmembrane 4 beta
ULK1: Unc-51 like kinase-1
LKB1: Liver kinase B1
AMPKα1: AMP-activated protein kinase alpha 1
LC3B: Microtubule-associated protein 1 light chain 3 B
Atg14: Autophagy Related 14
PI3K: Phosphoinositide 3-kinase
PTEN: Phosphatase and tensin homolog
GFP: Green fluorescent protein
RFP: Red fluorescent protein
AICAR: 5-aminoimidazole-4-carboxamide riboside
CRISPR/Cas9: Clustered regularly interspaced short palindromic repeats/ CRISPR-associated endonuclease
