# Supplementary material for: Sushi domain-containing protein 4 binds to epithelial growth factor receptor and initiates autophagy in an EGFR phosphorylation independent manner
Source: J Exp Clin Cancer Res. 2022 Dec 29;41:363. doi: 10.1186/s13046-022-02565-1 (PMC9798675; doi:10.1186/s13046-022-02565-1)
Supplement: Supplementary file 1 — Additional file 1. [file 13046_2022_2565_MOESM1_ESM.pdf]

Figure S1

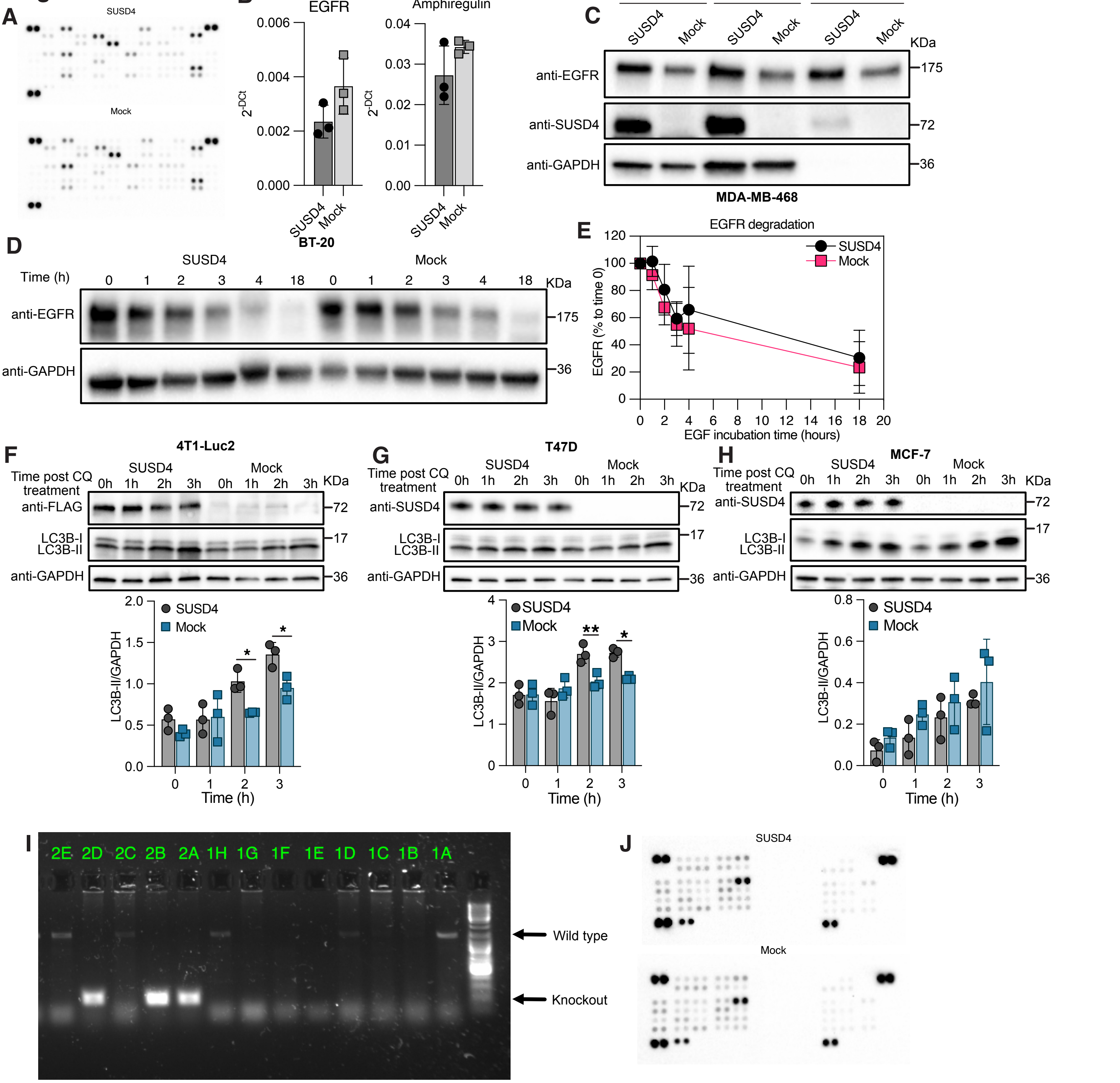

Fig. S1. Effect of SUSD4 expression on signaling molecules. (**A**) Dot plots image of the Protein profile Human XL Oncology Array (**B**) Quantitative real-time PCR comparing the expression of EGFR and amphiregulin between BT-20 cells stably expressing SUSD4 and mock control cells. (**C**) Biotinylation of cell surface proteins in MDA-MB-468 cells stably expressing SUSD4 and mock control cells followed by immunoblot detection of SUSD4 and EGFR in the intracellular- and plasma membrane fractions.EGFR degradation in SUSD4-expressing BT-20 cells and mock control cells. Representative immunoblots (**D**) and densitometric analysis of EGFR levels relative to baseline (**E**) following serum starvation and treatment with EGF. Evaluation of LC3B-II levels under three hours of chloroquine treatment for 4T1Luc2 (**F**), T47D (**G**), and MCF-7 (**H**) cells stable expressing SUSD4 compared with the control mock cells. (**I**) A representative image of agarose gel with PCR products of amplified fragment of the exon 8 to exon 10 of EGFR after CRISPR/Cas9 genome editing of the BT-20 breast cancer cell line. (**J**) Dot plots image of the Human Phospho-Kinase Antibody Array. All results were repeated in at least three independent biological experiments. The p-value was estimated with t test when two groups were compared and with Two-way ANOVA test when two or more groups were compared for two independent parameters, *p≤0.05, **p≤0.01, ***p≤0.001 and ****p≤0.0001.

Figure S2

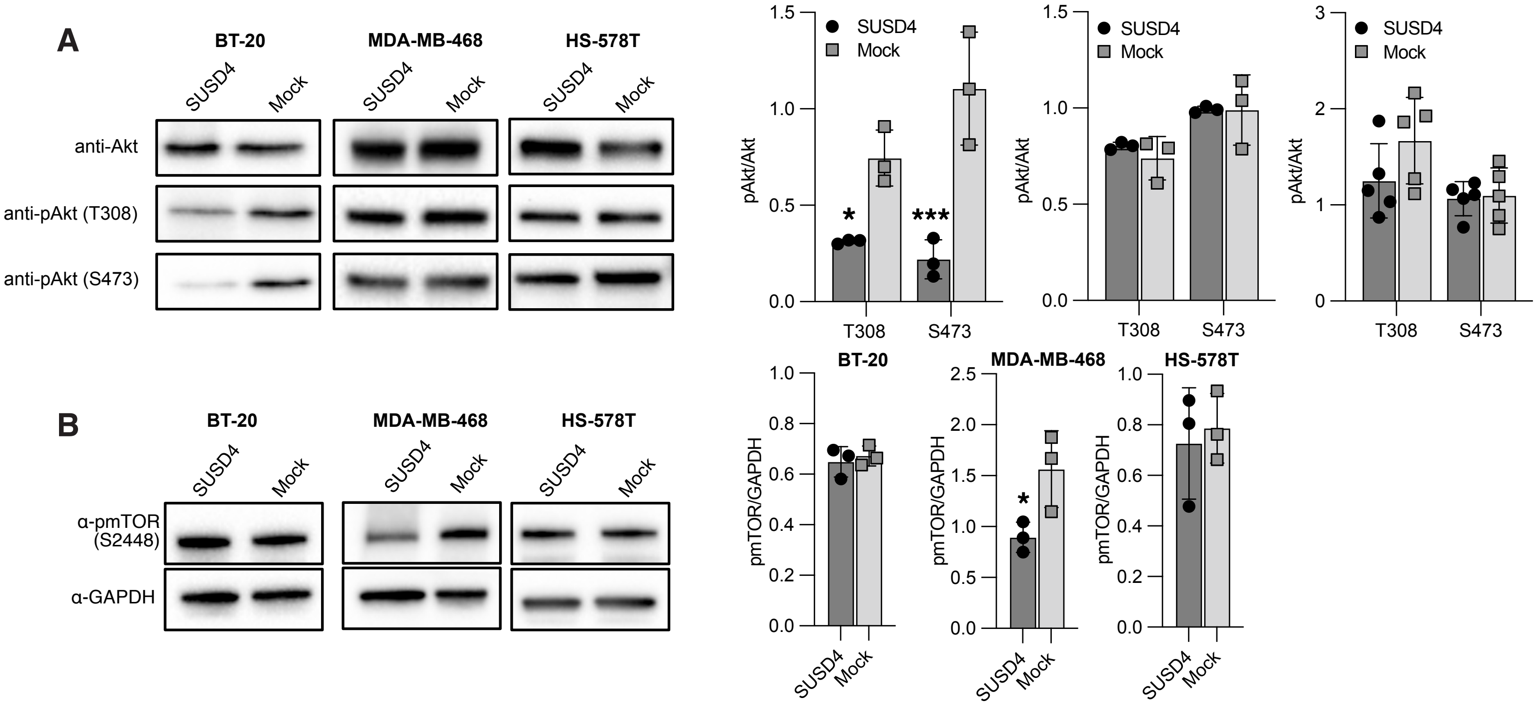

Fig. S2. (**A**) Representative immunoblots and densitometric analysis showing the ratio of phosphorylated Akt to total Akt in BT-20 cells, MDA-MB-468 cells and HS-578T cells stably expressing SUSD4 and the corresponding mock control cells. Two distinct phosphorylation sites were assessed (Tyr308 and Ser473) for BT-20, MDA-MB-468 and HS-578T cells. (**B**) Representative immunoblots and densitometric analysis of phospho-mTOR (Ser2448) levels in SUSD4-expressing BT-20-, MDA-MB-468-, and HS-578T cells and the corresponding mock control cells. All results were repeated in at least three independent biological experiments. The p-value was estimated with Two-way ANOVA test $^{*}p \leq 0.05$, $^{**}p \leq 0.01$, $^{***}p \leq 0.001$ and $^{****}p \leq 0.0001$.

Figure S3

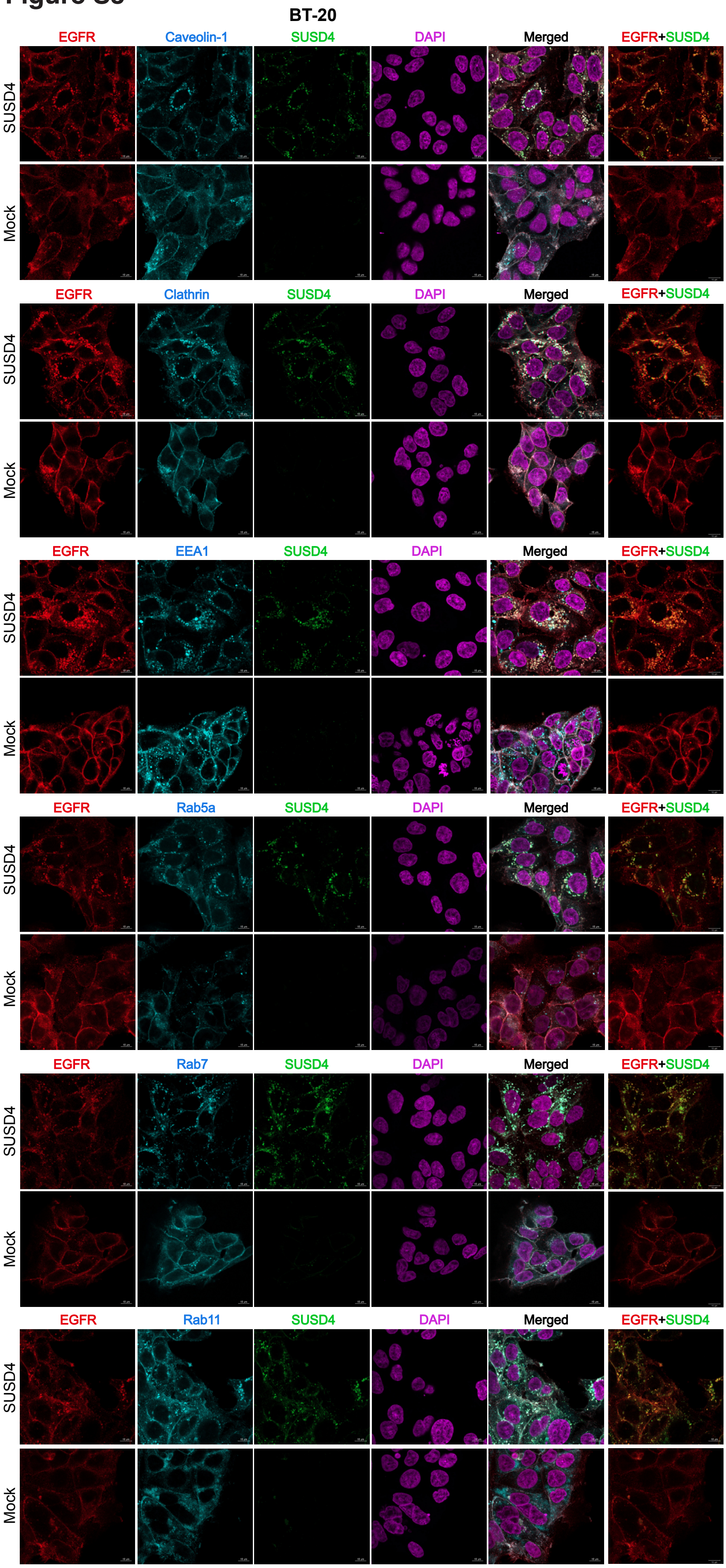

Fig. S3. Colocalization of SUSD4 and EGFR with endo-/lysosomal markers. Representative single-channel and merged confocal images co-stained for EGFR (red), SUSD4 (green), endo-/lysosomal markers (orange) and DAPI (blue) in SUSD4-expressing BT-20 and corresponding mock control cells. All results were repeated in at least three independent biological experiments

Figure S4

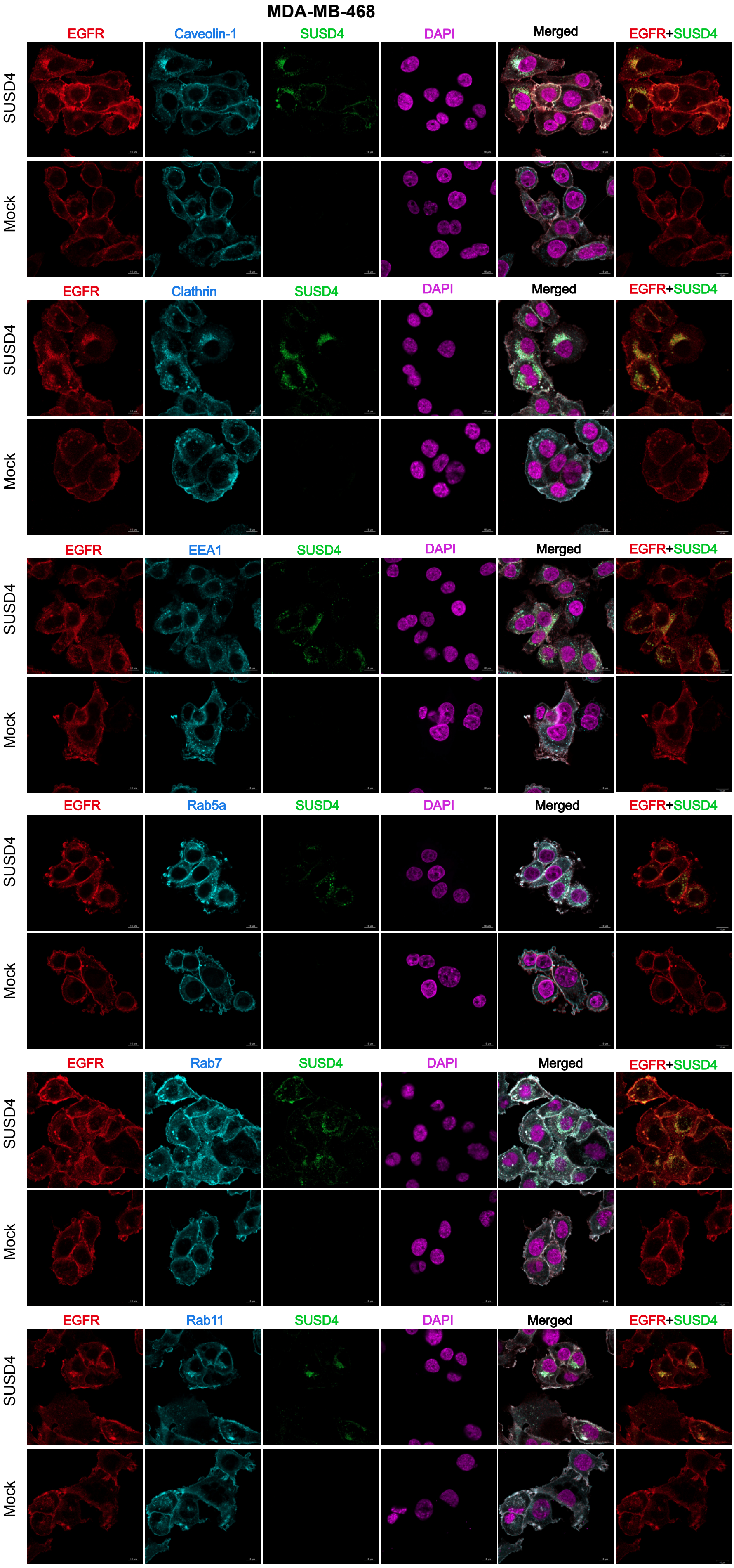

Fig. S4. Colocalization of SUSD4 and EGFR with endo-/lysosomal markers. Representative single-channel and merged confocal images co-stained for EGFR (red), SUSD4 (green), endo-/lysosomal markers (orange) and DAPI (blue) in SUSD4-expressing MDA-MB-468 cells and corresponding mock control cells. All results were repeated in at least three independent biological experiments

Figure S5

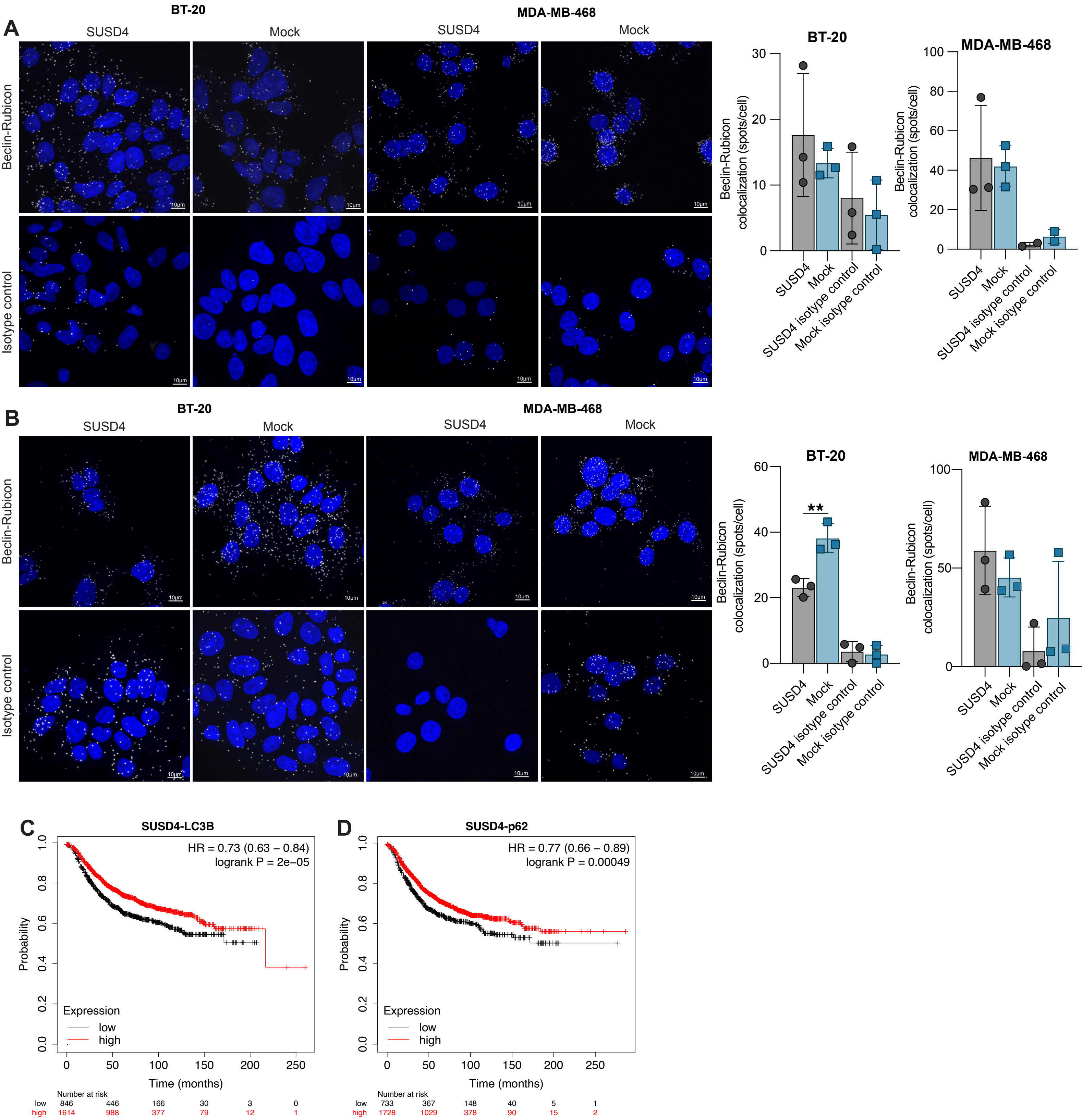

Fig. S5. Assessment of colocalization between Rubicon and Beclin-1. (A) Representative confocal images from a proximity ligation assay and quantified colocalization (white spots) of Rubicon and Beclin-1 in SUSD4-expressing BT-20 cells or MDA-MB-468 cells and corresponding mock control cells. **(B)** Same as **(A)** but cells were serum-starved prior to fixation for the proximity ligation assay. Kaplan Meier survival analysis showing relapse free survival for breast cancer patients with high or low levels of SUSD4 expression and positive for the expression of autophagy markers LC3B (**C**) and p62 (**D**). All results were repeated in at least three independent biological experiments. The p-value was estimated with t test when two groups were compared *p0.05, **p0.01, ***p0.001 and ****p0.0001

Table S1. Plasmids constructed and used in this study.			
**Plasmid name**	**Backbone**	**Insert**	**Resistance**
pCDNA3+SUSD4	pCDNA3	cDNA human SUSD4	Ampicillin, G418
pCDNA3+mSUSD4	pCDNA3	cDNA mouse SUSD4	Ampicillin, G418
pCDNA3+SUSD4ΔCCP1	pCDNA3	cDNA human SUSD4 (del. 169-357)	Ampicillin, G418
pCDNA3+SUSD4ΔCCP2	pCDNA3	cDNA human SUSD4 (del. 361-537)	Ampicillin, G418
pCDNA3+SUSD4ΔCCP3	pCDNA3	cDNA human SUSD4 (del.538-717)	Ampicillin, G418
pCDNA3+SUSD4ΔCCP4	pCDNA3	cDNA human SUSD4 (del. 718-912)	Ampicillin, G418
pCDNA3+SUSD4LSPF	pCDNA3	cDNA human SUSD4 Y379F	Ampicillin, G418
pCDNA3+SUSD4PPAF	pCDNA3	cDNA human SUSD4 Y414F	Ampicillin, G418
PX459+sgRNAEGFREx8F	PX459	GTGTGTAACGGAATAGGTAT	Ampicillin, Puromycin
PX459+sgRNAEGFREx10F	PX459	ACCTAGAAATCATACGCGGC	Ampicillin, Puromycin
EGFR-KD	pEGFP-N1	cDNA human EGFR-EGFP K721A	Kanamycin

Table S2. Primers used in this study.		
**Name**	**Sequence (5'-3')**	**Description**
ΔCCP1F	TCACGGGCGGGTTCGATGACCTTGATTGCCGTATCCCTCAAATCGAAGATG	del. 169-357
ΔCCP2F	AGTGATAATTCCATCTGTGTGCAAGAATGCCTGAGACCTCTAGCCTCT	del. 361-537
ΔCCP3F	TGGAATAATCTGCCCATCTGTCAAGGCCTGGAAGTCTGTCCACTACCTCCAATG	del.538-717
ΔCCP4F	GCCCACCCCGGTGCCTTGCTTCAGAGCAAACGTGGCCCAGCA	del.538-717
LSPYmutF	GACGGCGTGCCCGTCATGCTCCCGTCCTTTGACGAAGCTGTGAGTGGCGGCTTGAGTG	SUSD4 Y379F
PPAYmutF	GGACGACCAGAGCCCCCCAGCATTCCCCGGCTCAGGGGACACGGACACAG	SUSD4 Y414F
EGFR-KDF	GGGTGAGAAAGTTAAAATTCCCGTCGCTATCGCGGAATTAAGAGAAGCAACATCTCCGAAAG	EGFP K721A
EGFR Exon8F	TCCAACAAATGTGAACGGAATA	EGFRKO-PCR
EGFR Exon10R	TGCCCTATCTTAGCAACTCTCC	EGFRKO-PCR

Table S3. List of antibodies			
**Target**	**Subtype**	**Supplier**	**Catalogue number**
SUSD4	Polyclonal Rabbit IgG	Homemade	-
EGFR	Polyclonal Goat IgG	R&D systems	AF231
Phospho-EGFR (Tyr1045)	Polyclonal Rabbit IgG	CST	2237
Phospho-EGFR (Tyr1086)	Monoclonal Rabbit IgG	R&D systems	MAB8967
GAPDH	Mouse monoclonal IgG1	Abcam	ab8245
Phospho-Akt (Ser473)	Monoclonal Rabbit IgG	CST	4060
Phospho-Akt (Thr308)	Monoclonal Rabbit IgG	CST	13038
Akt	Monoclonal Rabbit IgG	CST	4691
Mouse IgG HRP	Polyclonal Goat IgG	Dako	P0447
Rabbit IgG HRP	Polyclonal Goat IgG	Dako	P0448
Rabbit IgG HRP	Polyclonal Goat IgG	CST	7074
AMPKα1	Monoclonal Rabbit IgG	CST	5831
Phospho- AMPKα1 (Thr172)	Monoclonal Rabbit IgG	CST	2535
Phospho-ULK1 (Ser757)	Polyclonal Rabbit IgG	CST	6888
mTOR	Monoclonal Rabbit IgG	CST	2983
Phospho-mTOR (Ser2448)	Monoclonal Rabbit IgG	CST	5536
PDGFRα	Monoclonal Mouse IgG1	R&D systems	MAB322
LC3B	Polyclonal Rabbit IgG	Sigma	L7543
Anti-SQSTM1/p62	Mouse monoclonal	Abcam	ab56416
ULK1	Monoclonal Rabbit IgG	CST	8054
Phospho-ULK1 (Ser757)	Polyclonal Rabbit IgG	CST	6888
Phospho-ULK1 (Ser555)	Monoclonal Rabbit IgG	CST	5869
Phospho-ULK1 (Ser317)	Polyclonal Rabbit IgG	CST	37762
Beclin-1	Monoclonal mouse IgG1	CST	4122
phospho-Beclin-1 (Ser30)	Monoclonal Rabbit IgG	CST	35955
Atg14	Monoclonal Rabbit IgG	CST	96752
Phospho-Atg14 (Ser29)	Monoclonal Rabbit IgG	CST	92340
LKB1	Monoclonal Rabbit IgG	CST	3047
Phospho-LKB1 (Ser428)	Monoclonal Rabbit IgG	CST	3482
Caveolin-1	Monoclonal Rabbit IgG	CST	3267
Clathrin Heavy Chain	Monoclonal Rabbit IgG	CST	4796
EEA1	Monoclonal Rabbit IgG	CST	3288
Rab5A	Monoclonal mouse IgG1	CST	46449
Rab7	Monoclonal Rabbit IgG	CST	9367
Rab11	Monoclonal Rabbit IgG	CST	5589
